# Plant Variety Protection: Current Practices and Insights

**DOI:** 10.3390/genes12081127

**Published:** 2021-07-25

**Authors:** Ju-Kyung Yu, Yong-Suk Chung

**Affiliations:** 1 Syngenta Crop Protection LLC, Seeds Research, Triangle Research Park, NC 27709, USA; ju-kyung.yu@syngenta.com; 2 Department of Plant Resources and Environment, Jeju National University, Jeju 63243, Korea

**Keywords:** breeding, international union for the protection of new varieties of plants (UPOV), plant variety protection (PVP), breeder’s right, DUS test (distinctness, uniformity, and stability), essentially derived variety (EDV)

## Abstract

Breeders persistently supply farmers with the best varieties in order to exceed consumer demand through plant-breeding processes that are resource-intensive. In order to motivate continuous innovation in variety development, a system needs to provide incentives for plant breeders to develop superior varieties, for example, exclusive ownership to produce and market those varieties. The most common system is the acquisition of intellectual property protection through plant variety protection, also known as the breeder’s right. Most countries have adopted the system established by the International Union for the Protection of New Varieties of Plants (UPOV). To be granted plant variety protection, the variety should prove to be unique by meeting three requirements: distinctness, uniformity, and stability. This review summarizes (1) the plant variety protection via UPOV convention, (2) technical methods for distinctness, uniformity, and stability testing via phenotype, molecular markers, and sequencing as well as their challenges and potentiality, and (3) additional discussions in essentially derived variety, value for cultivation and use testing, and open source seed initiative.

## 1. Introduction

Plant varieties can be improved for numerous agronomic reasons, such as better yields, quality, and resistance to biotic and abiotic stresses. In modern agriculture, crop varieties are derived through plant breeding by mating two or more parental lines that contain desirable characteristics, and the target characteristics are measured over multiple generations under different environmental conditions. Among the progeny, individuals with desirable characteristics are selected, whereas individuals with undesired characteristics are eliminated from the breeding process. This process, repeated over many generations, can create favorable combinations of genetic variations in the next generation, resulting in superior varieties [1,2].

The general plant breeding process for both inbreeding and hybrid crops begins with large populations of candidate lines. For example, a commercial corn breeder in the US typically screens approximately 1000 individuals to generate 10–15 individuals in order to introgress the desired characteristic into one competitive genetic background. The 10–15 selected parental plants are cross-pollinated to generate more than 150,000 progeny plants that are evaluated for numerous agronomic characteristics over the course of approximately 6–7 years [1,3,4]. More than 99.9% of the progeny plants are eliminated to identify commercially competitive varieties that meet market demand [3,4]. A breeder usually introgresses several characteristics into multiple backgrounds that have adapted to different environmental conditions. Therefore, this process can result in the screening of hundreds of thousands of individual plants that are grown in hundreds of different environments [3]. Currently, marker-assisted selection can be applied as a two-step process: (1) select progeny carrying the favorable characteristics using markers (trait markers) and (2) screen and select progeny with those favorable genetic backgrounds using markers (genome-wide markers) [1,3,5].

Breeding new varieties is a resource-intensive activity in terms of costs, infrastructure, genetic resources, and the breeders’ knowledge and experience. It can also be time-consuming: it typically takes around six to seven years to release a new variety but can take up to ten years [1,2,5]. A video recording on the costing of breeding programs in the public and private sectors can be viewed at https://www.youtube.com/watch?v=x8QXauBzhYo, accessed on 21 July 2021. However, plant varieties can be reproduced easily and quickly, and breeders need to secure their investment return. Plant breeders have various options to protect their ownership of new varieties, prevent outlawed practices and recover breeding costs from royalties. Such options motivate breeders and support further breeding activities that constantly provide farmers with the best varieties that satisfy consumer demand [3,4,6,7].

## 2. Protection of the Breeder’s Right

Plant variety protection (PVP) via the International Union for the Protection of New Varieties of Plants (UPOV) is a harmonized system that awards intellectual property rights (IPR) to organizations in its 75 member states (as of February 2019; https://www.upov.int/portal/index.html.en, accessed on 21 July 2021). It recognizes both the plant breeder’s right (PBR) and the plant variety right (PVR). Under the 1991 Act of the UPOV Convention, the PBR is granted for a period of no less than 20 years [8,9].

Under the UPOV system, anyone can be a breeder and can apply for PVP. However, a new plant variety can only be protected if it meets four criteria established by the UPOV: (1) it must be a novel variety, (2) it must be distinct from other varieties, (3) it must display homogeneity, and (4) it must be stable, remaining unchanged from one generation to the next [8,9]. Only the breeder can obtain protection for a new variety. A few examples of activities that can be considered infringements if they are carried out without the certified owner’s permission are (1) the sale and marketing of a protected variety, (2) sexual multiplication or unauthorized propagation, (3) use of parental lines for hybrid variety production, and (4) distribution of the variety without the permission of the certified owner. Under the 1991 Act of the UPOV Convention, there are a few exceptions to the breeder’s right, also known as breeder’s exemption: the right of all to use protected varieties as sources of initial variation, the right of all to use protected varieties for research or experimental purposes, and the right of all to use protected varieties for private, non-commercial purposes (e.g., amateur gardeners) [8,9].

In some countries, such as the USA, Japan, and Australia, breeders can also apply for plant or utility patent rights if the new varieties satisfy the required criteria. The Plant Variety Protection Office (PVPO) in the US provides intellectual property protection for breeders of new varieties of seeds, tubers, and asexually propagated plants (https://www.ams.usda.gov/services/plant-variety-protection, accessed on 21 July 2021). There are three types of intellectual property protection: (1) PVP issued by the PVPO, (2) plant patents issued by the Patent and Trademark Office (PTO) for asexually propagated plants (except edible tubers), and (3) utility patents issued by the PTO for genes, traits, methods, plant parts, or varieties [7]. This article will focus on PVP under the UPOV system. The authors will seek another opportunity to address different types of PVP, such as variety patents, utility patents, trade secrets, and more.

These protection systems aim to give the owners of plant varieties exclusive rights to control production, distribution, and marketing and assure that owners are compensated for the costs of developing new varieties.

## 3. The Distinctness, Uniformity, and Stability (DUS) Test

**Figure 1:**
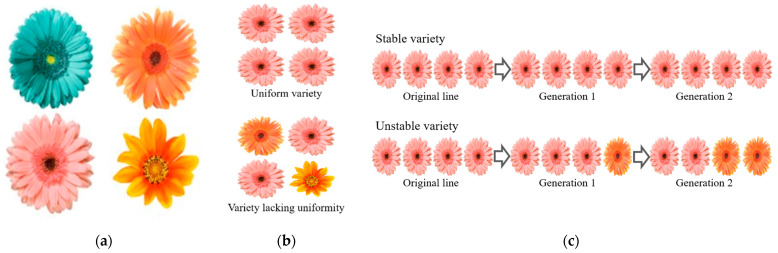
Illustration of DUS definitions. (**a**) Distinctness; (**b**) Uniformity; (**c**) Stability.

Within the UPOV’s PVP framework, all members acknowledge breeders of new varieties of plants by granting them intellectual property rights based on the following conditions: distinctness (D) from existing varieties, uniformity (U) of relevant characteristics that depend on the reproduction system of the species, and stability (S) in expression of traits after two independent growing cycles (Figure 1). Details of these conditions can be accessed from https://www.upov.int/overview/en/conditions.html, accessed on 21 July 2021. The system for establishing distinctness, uniformity, and stability is known as the DUS test (UPOV 1991) [8,9]. Therefore, before applying for PVP, applicants should have bred a new variety, completed the DUS test, and chosen a unique name for it [5,7,8]. The following sections discuss methods of proving DUS—phenotypes-based, molecular markers-based, and sequencing-based tests—and their limitations, practicable solutions, and potentialities.

## 4. Phenotypes-Based DUS Test

The current PVP system relies on the morphological description of plant varieties. The DUS test determines whether a new variety is distinguishable from commonly known varieties and exhibits sufficient phenotypic uniformity and stability. A DUS test is normally conducted in a field or greenhouse over two successive growing seasons at two test locations. During this period a number of morphological characteristics are recorded for both the new (or candidate) variety and similar varieties, in what is known as *common knowledge*. The DUS test, whether it is carried out in a trial field or greenhouse, must follow internationally agreed protocols and UPOV guidelines [8,9]. Over 300 test guidelines are currently available for different plant species; these guidelines can be accessed from the UPOV website at https://www.upov.int/resource/en/dus_guidance.html, accessed on 21 July 2021. Table 1 shows the list of characteristics used for testing corn, wheat, rice, barley, and soybean among others.

The phenotype-based DUS test is a time-consuming, laborious (frequently requires skill and adequate experience), and costly process of field and greenhouse assessment [5,10,11,12]. In some cases, special conditions or trial designs are required to conduct the tests, for example, vernalization in barley and wheat. In addition, as the numbers of candidates and publicly known cultivars increase, the ability to distinguish them on the basis of morphological traits alone becomes more difficult, even though differences in agronomic performance may exist.

## 5. Limitations of the Conventional Phenotypes-Based DUS Test Results

In the current system, many factors limit the use of morphological traits to conduct a DUS test. The measurement of morphological traits may be influenced by environmental factors, including locations and time spans, which may complicate the assessment [11,12]. These factors can affect the expression of morphological traits, resulting in reduced precision and discriminatory power. This is especially disadvantageous for the evaluation of disease-resistant traits, which are mandatory for the majority of crops, or complex traits (known as quantitative traits), such as drought resistance and flowering time [11,12,13]. 

In a backcross breeding program, the top-performing variety (existing protected variety) is often repeatedly crossed with other breeding lines. This requires ongoing evaluation and selection of progeny with the most desired agronomic characteristics and removal of any progeny with undesired characteristics. By the sixth backcross, the selected progeny will theoretically contain more than 99% of the genetic background of the top-performing varieties, but less than 1% of the other breeding lines [1,3,4]. There are other breeding techniques, such as mutation breeding and genetic engineering, that can also create minor genetic modifications [14]. In these cases, a new variety would be considered to be an essentially derived variety (EDV) from the initial variety (IV) [14,15]. The essential derivation and dependency concepts have also been defined by the 1991 Act of the UPOV Convention; with this process, most of the IV’s essential characteristics remain intact in the newly bred variety [8,9]. For example, if a new variety meets the criteria of practically containing the entire genotype of a variety from which it was derived, and therefore, genetically maintains the expression of the IV’s essential traits, the newly bred variety is considered to be an EDV from the IV. Therefore, EDVs share their essential traits with the IV, except their distinctive traits. To determine EDV, or distinguish between EDV and fraud, the criteria and procedures used for phenotype-based DUS tests are often insufficient or not applicable because there will always be important morphological traits that differentiate an EDV from the IV [15]. 

The increased availability and cost-efficiency of molecular markers make them appealing options that supplement existing phenotype-based DUS tests. Molecular markers-based DUS tests have several notable advantages over phenotype-based DUS tests, such as high discriminatory power, high repeatability, independence from environmental effects, applicability to seed or early growth stages of plants, rapid data production and analysis, and amenability to readily searchable databases containing records of thousands of varieties, which can also facilitate global harmonization [16]. In addition, to characterize EDVs, molecular markers can be used to conduct tests based on the genetic relatedness of IVs and EDVs [15].

## 6. Molecular Markers-Based DUS Test

The UPOV’s Working Group on Biochemical and Molecular Techniques and DNA-Profiling in Particular (BMT) consists of biochemical and molecular DUS testing experts and plant breeders (UPOV 2013; https://www.upov.int/edocs/tgpdocs/en/tgp_15.pdf, accessed on 21 July 2021). The purpose of the UPOV-BMT is to explore practical aspects, such as the application of molecular markers for plant-variety registration, and to strengthen the protection afforded by the PBR. So far, the BMT has proposed two models. Model 1 is the use of molecular markers that are directly linked to traditional and molecular characteristics as predictors of traditional characteristics. It requires prerequisite research to identify marker–trait associations that maintain their robustness across diverse germplasm [17]. Model 2 is the calibration of threshold levels for molecular markers against the minimum distance in phenotype traits. This application potentially provides the basis for the introduction of a system for combining phenotypic and molecular distances in the management of variety collections as a tool to improve the efficiency of distinctness evaluation [18,19].

Due to the abundance of reference genomes and advances in molecular technologies, such as gene cloning, markers that target the specific alleles responsible for phenotype variation, the so-called functional markers (FM), can now be developed [20]. FMs could be highly predictive of trait expression and overcome the problem of using flanking markers to the traits that are prone to recombination between the marker and the actual target alleles.

Various studies have demonstrated the use of markers for the assessment of distinctness [17,21,22,23,24,25,26,27]. Cockram et al. [17] tested 25 single nucleotide polymorphism (SNP) markers for 14 DUS traits on a panel of 169 barley varieties. Three DUS traits (ear (number of rows), grain (disposition of lodicules), and seasonal growth habit) were perfectly predicted by the genotype data. The remaining traits, ranging from 81% to 91%, were also predicted by the genotype data. With 41 corn inbred lines, simple sequence repeat (SSR) markers showed better performance than DUS traits in distinguishing and fitting their pedigree information [27]. Sarao et al. [28] demonstrated that molecular-marker data differentiated japonica and indica rice subspecies. SSR markers, using cluster and principal component analysis (PCA), distinguished basmati from non-basmati groups and showed congruency with the pedigree and breeding history of 14 rice varieties [28]. These results suggest that the prediction value and genetic distance calculated on the markers revealed better consistency and correlated with the pedigree of varieties. 

Wang et al. [29] established a method of applying molecular markers for the assessment of stability. A set of 347 SSR markers was genotyped using 20 wheat breeding lines and the homozygous SSR loci ratio (SSR–HLR) was calculated to identify non-homozygous SSR loci. By comparing the morphological observation with the SSR-HLR, more than 95% of varieties were considered stable, and less than 91% were considered unstable. The varieties with SSR–HLR ranging from 91% to 95% were suggested for morphological observations as the field condition for stability assessment. This approach has several advantages: better selection of relevant references in field trials, exclusion of non-relevant reference varieties from field trials, reduction in the number of reference varieties in field trials, and reduction in the duration of field trials, which leads to a reduction in overall costs and resources [29,30,31].

In cases where molecular data provide greater discrimination than phenotypic comparisons, an important question needs to be asked when using molecular markers in DUS tests: how different is different? Breeders have mentioned that the use of marker data in DUS evaluation might lead to the introduction of unrealistically and unnecessarily high levels of uniformity being required at the DNA sequence level, which may lead to higher resource demands during breeding and seed multiplication [5,13]. Regardless of data sources, whether morphological, physiological, or molecular markers, determining a distinctness threshold may lead to the question of how to define minimum distance in order to maintain the current level of intellectual property protection [32,33,34].

Archard et al. [13] compared the SNP-based similarities and pedigree-based kinships of 322 closely related PVP-certified soybean varieties. The initial analyses suggest there is evidence of distinctness where SNP similarity is in the range of 93–97%. A subsequent analysis suggests that 96% SNP similarity could provide an acceptable threshold for the assessment of distinctness. Consequently, varieties that are more than 96% similar according to SNP marker data, but also differ in their morphological or physiological traits, would continue to be classified as distinct for as long as these traits are the ultimate test of distinctness. Archard et al. [13] measured the intracultivar heterogeneity of SNP loci of 35 commercially developed soybean varieties. The levels of intracultivar SNP heterogeneity were low (range 0–5%, mean 1.8%, and standard deviation 1.3%) for all 35 varieties. These levels of SNP heterogeneity are consistent with the commonly used breeding strategy of bulking individual plants at the F4 stage of inbreeding. 

Van Eeuwijk and Law [35] discussed the criteria to select markers when developing an EDV protocol, including marker unbiasedness, precision, and genome coverage. The distribution of genetic similarities between corn parental inbred lines and their progeny derived from F2 and different backcross populations was examined to determine whether markers should be evenly distributed across the entire genome or be confined to specific regions that control differences in DUS traits. The results show that increasing the number and length of chromosomes when using abundant and evenly distributed markers leads to a decrease in standard deviations, which in turn increases prediction accuracy.

## 7. Sequencing-Based DUS Test

There are technical challenges for molecular markers-based assessment. Even though functional markers (FM) are becoming more abundant, the majority of molecular markers applied to breeding programs are not directly linked to traits. Certain genomic areas of trait that are of interest are located in highly repetitive genome areas, which makes the design of markers difficult. In addition, there are other types of genomic variants, such as structural variants (SVs) and copy number variations (CNV) that are also directly responsible for traits. Identification of such causative variants has been inhibited due to the limitations of common marker technologies, such as SNP and SSR. Due to the rapid cost reductions of next-generation sequencing (NGS) and genotyping-by-sequencing (GBS) platforms, plant breeders are switching from conventional marker technology to sequencing technology [36,37,38,39]. Various NGS technologies are being applied to breeding programs, for example, short-read whole-genome sequencing (WGS), and targeted NGS or targeted GBS. 

Targeted NGS allows the sequencing of a specific genomic region in order to detect causative variants more rapidly and cost-effectively than WGS. Targeted NGS may become an attractive replacement for the conventional marker technology that is used for marker-based DUS testing [39]. CRISPR-Cas9 -enriched long-read sequencing (LRS) is a good example of targeted NGS. By directing CRISPR-Cas9 for targeted cleavage in order to isolate a region of interest, followed by enrichment and LRS, it is possible to excise long, intact DNA molecules from the trait of interest [37,38]. This allows the target region to be isolated without amplification and subsequently investigated using LRS technologies. The resulting long-read data not only give a comprehensive view of the region of interest and make it possible to identify complex structural variation but also repeat elements that are difficult to find with other DNA-sequencing technologies.

## 8. Noteworthy Subjects in Addition to the Current UPOV and DUS Tests

By definition of the UPOV convention, conventional breeding generally relies on genetic recombination. The process enables the selection of superior varieties by creating new allelic and phenotypic combinations. With advances in new breeding technologies (NBTs), plant breeders could manipulate gene sequences precisely in order to create new favorable alleles or remove unfavorable ones [14]. This technology, known as genome editing, may revolutionize plant breeding, leading to the development of new varieties [40,41]. Among the various genome editing tools, CRISPR is becoming popular because of its high efficiency and simplicity [41,42,43]. It modifies genotypic distances between varieties through genome editing, and the modification can occur at the level of a few base-pair nucleotides. However, this leads to some controversy. If the IV is the variety protected by the PBR, the new genome-edited variety would be subject to an EDV claim since it wholly conforms and retains the essential characteristics of the IV except for a few base-pair nucleotides. This minor modification adds significant agronomic value since herbicide resistance by the IV could also be considered a distinctive trait in DUS testing. Legal clarification on the definition of EDV is of fundamental importance in order to avoid or discourage active adaption of NBT in breeding programs. Therefore, redefinition of the EDV concept is inevitable, and harmonized guidelines on what is publicly and internationally accepted are required by next-generation breeders [16,44]. 

In the EU, a mandatory variety-testing system is used in addition to DUS testing before new varieties are released (value for cultivation and use (VCU) testing). Where DUS traits focus on morphological characteristics, VCU focuses on more agronomic characters [45,46,47]. There are four categories for VCU testing: (1) yield; (2) resistance to biotic and abiotic stresses; (3) end-use quality, such as malting quality in barley, protein content in wheat, and oil quality/quantity in rapeseeds; and (4) factors in the physical condition, such as susceptibility to damage. Some candidate varieties with proven superior VCU fail DUS even though the non-distinct comparison is made with a significantly lower-performing registered cultivar. To resolve this case, an additional value molecular-linked DUS (vmDUS) distinctness tool that uses molecular markers, but conforms to UPOV-declared principles, has been proposed [47].

The open source seed initiative (OSSI) was initiated in 2012, inspired by the open source software movement [48,49,50]. The OSSI emerged in the United States, where patenting of plant varieties is common. OSSI wants to create a system that can go viral: IVs would be freely available to breeders under the condition that any genetic resources (varieties) derived from them would be made available under the same open source conditions. This would be achieved through a *pledge* (in the USA). The F1 hybrid can be OSSI-pledged with the information from parental lines involved in the cross to produce the hybrid, but the parental lines will not be required to be OSSI-pledged or available (https://osseeds.org/faqs/, accessed on 21 July 2021). In 2014, 37 varieties of 14 species were released under the OSSI-Pledge by various breeders in the public and private sectors. PVP has been developed in order to stimulate invention and is intended to support the use of inventions in practical innovations that positively impact society [48,49]. There is some concern that the OSSI movement may discourage innovation and that it may eventually negatively impact societal benefits. Finding a balance between breeders’ rights and obligations is complex in a society where rapidly advancing technology increasingly alienates the inventor from society at large [50]. OSSI should consider a way of providing sufficient innovation that requires significant investments in plant breeding that contribute to societal goals, including the provision of more robust plant varieties that meet farmers’ and end-user customers’ demands.

## 9. Closing Remark

Advances in genotyping and sequencing platforms, and reductions in their usage costs, provide opportunities for their potential application in variety registration. Chip and array technologies, consisting of hundreds of thousands of SNP markers, are being actively applied to breeding programs [51,52,53,54]. In addition, advances in statistical methodologies and increased computing power have led to genome-wide, low-passing sequencing technology being rapidly adopted by breeding programs [55]. However, none of these have been officially adopted by the UPOV. The UPOV currently requires the use of molecular markers only when they perfectly correlate with DUS traits, which does not reflect the advances in genotyping and sequencing technologies or an understanding of DUS trait genetics. Authors should urge the BMT working group to kick off another round of technical discussions related to the recognition of the potential of these technologies, the exploration of new opportunities, and the prompt adoption of these technologies for next-generation DUS testing.

Herein, the overall cost estimation of PVP, including DUS testing, is not addressed. One of the main reasons is that the current breeding operation adoption level and molecular technologies vary by public vs. industry, well-funded institutes vs. poorly funded, and major grain crops vs. vegetables. For example, if a breeder works in a well-funded institute or industry, all DUS characteristics and molecular marker information can be gathered during the line development stage. Then, this breeder (or institute) may only need resources to cover the cost of registration and maintenance of the PVP. However, if a breeder works in an older or poorly funded breeding environment, the cost of gathering DUS characteristics is incredibly challenging. Therefore, the authors consider that cost estimation of the PVP process may not be relevant and desire to avoid any possibility of misinterpretation of the information by readers.

As discussed, new breeding technologies, such as genome editing, are common in both the public and private sectors. Consideration of a new definition of plant variety is a priority for those involved in the development, evaluation, and release of new crop varieties. Plant variety patenting is common practice in the US, for example. VCU testing is now a mandatory step, in addition to DUS testing, before variety registration in the EU. Procedures and technical methods for plant variety testing require significant, harmonized international standards that create the basis for strengthening the supply of improved varieties and the PVP system. Therefore, as an intergovernmental organization, the UPOV’s direct involvement and significant contribution to these areas are urged.

When a PVP certificate officially expires (exPVP), and the active variety patent is no longer valid, the original variety enters the public domain. With advanced high-throughput, high-density genotyping and sequencing technologies combined with advanced statistical mythologies and high computing power, exPVP lines potentially represent a new germplasm source for any breeding program [56,57]. It has also opened new opportunities to discover and exploit the genes controlling agronomic performance in conventional breeding programs and genome editing programs. As mentioned earlier, the authors plan for another manuscript that will focus on variety protection beyond PVP under the UPOV system, like variety patents, utility patents, trade secrets, and how public and private sectors utilize exPVP lines for their breeding and research programs.

## Figures and Tables

**Table 1:** The list of DUS traits required by UPOV convention: barley, corn, rice, soybean, and wheat as examples.

Barley	Cron	Rice	Soybean	Wheat
Kernel: color of aleurone layer	Leaf: angle between blade and stem	Leaf: intensity of green color	Hypocotyl: anthocyanin colorant	Seed: color
Plant: growth habit	Leaf: attitude of blade	Leaf: anthocyanin coloration	Leaf: blistering	Seed: coloration with phenol
Plant: intensity of green color	Stem: anthocyanin coloration of brace roots	Leaf: distribution of anthocyanin coloration	Leaf: shape of lateral leaflet	Coleoptile: anthocyanin coloration
Lowest leaves: hairiness of leaf sheaths	Tassel: time of anthesis	Leaf: anthocyanin coloration of auricles	Leaf: size of lateral leaflet	Plant: growth habit
Flag leaf: intensity of anthocyanin coloration of auricles	Tassel: anthocyanin coloration at base of glume	Leaf blade: length	Leaf: intensity of green color	Plants: frequency of plants with recurved flag leaves
Flag leaf: attitude	Tassel: anthocyanin coloration of glumes excluding base	Leaf blade: width	Flower: color	Flag leaf: anthocyanin coloration of auricles
Time of ear emergence	Tassel: anthocyanin coloration of anthers	Flag leaf: attitude of blade (early observation)	Plant: color of hairs of main stem	Time of ear emergence
Flag leaf: glaucosity of sheath	Tassel: density of spikelets	Flag leaf: attitude of blade (late observation)	Plant: growth type	Flag leaf: glaucosity of sheath
Awns: anthocyanin coloration of tips	Tassel: angle between main axis and lateral branches	Time of heading (50% of plants with heads)	Plant: growth habit	Flag leaf: glaucosity of blade
Ear: glaucosity	Tassel: attitude of lateral branches	Male sterility	Plant: height	Ear: glaucosity
Ear: attitude	Ear: time of silk emergence	Lemma: anthocyanin coloration of keel (early observation)	Pod: intensity of brown color	Culm: glaucosity of neck
Grain: anthocyanin coloration of nerves of lemma	Ear: anthocyanin coloration of silks	Lemma: anthocyanin coloration of area below apex (early observation)	Seed: size	Lower glume: hairiness on external surface
Plant: length (stem, ear, and awns)	Leaf: anthocyanin coloration of sheath	Lemma: anthocyanin coloration of apex (early observation)	Seed: shape	Plant: length
Ear: number of rows	Tassel: length of main axis above lowest side branch	Spikelet: color of stigma	Seed: ground color of testa (excluding hilum)	Straw: pith in cross section
Ear: development of sterile spikelets	Inbred lines only: Plant: length	Non prostrate varieties only: Stem length (excluding panicle)	Seed: hilum color	Ear: density
Sterile spikelet: attitude	Hybrids and open pollinated varieties only: Plant: length	Stem: anthocyanin coloration of nodes	Seed: color of hilum funicle	Ear: length excluding awns and scurs
Ear: shape	Plant: ear placement	Stem: anthocyanin coloration of internodes	Plant: time of beginning of flowering	Ear: scurs or awns
Ear: density	Leaf: width of blade	Panicle: length of main axis	Plant: time of maturity	Ear: length of scurs or awns at tip of ear
Ear: length (excluding awns)	Ear: length without husk	Panicle: awns		Ear: color
Awn: length	Ear: diameter without husk	Panicle: distribution of awns		Ear: shape in profile
Rachis: length of first segment	Ear: shape	Panicle: length of longest awns		Apical rachis segment: area of hairiness on convex surface
Rachis: curvature of first segment	Ear: number of rows of grains	Spikelet: pubescence of lemma		Lower glume: shoulder width
Median spikelet: length of glume and its awn relative to grain	Ear: type of grain	Spikelet: color of tip of lemma		Lower glume: shoulder shape
Grain: rachilla hair type	Ear: color of top of grain	Panicle: attitude in relation to stem slightly drooping strongly drooping		Lower glume: length of beak
Grain: spiculation of inner lateral nerves of dorsal side of lemma	Ear: anthocyanin coloration of glumes of cob	Panicle: attitude of branches		Lower glume: shape of beak
Grain: type	Kernel: row arrangement	Panicle: exertion		Lower glume: area of hairiness on internal surface
Grain: hairiness of ventral furrow	Kernel: poppiness	Time of maturity		Seasonal type
Lemma: shape of base	Kernel: sweetness	Lemma: color		
Seasonal type	Kernel: waxiness	Grain: weight of 1000 fully developed grains		
	Kernel: opaqueness	Grain: length		
	Kernel: shape	Grain: width		
	Kernel: 1000 kernel weight	Decorticated grain: length		
		Decorticated grain: width		
		Decorticated grain: shape		
		Decorticated grain: color		
		Endosperm: type		
		Endosperm: content of amylose		
		Decorticated grain: aroma		
